# Non-Surgical Definitive Treatment for Operable Breast Cancer: Current Status and Future Prospects

**DOI:** 10.3390/cancers15061864

**Published:** 2023-03-20

**Authors:** Yuta Shibamoto, Seiya Takano

**Affiliations:** 1 Department of Radiation Oncology, Narita Memorial Proton Center, 78 Shirakawa-cho, Toyohashi 441-8021, Japan; 2 Medical Physics Laboratory, Division of Health Science, Graduate School of Medicine, Osaka University, 1-7 Yamadaoka, Suita-shi 565-0871, Japan; 3 Department of Radiology, Graduate School of Medical Sciences, Nagoya City University, 1 Kawasumi, Mizuho-cho, Mizuho-ku, Nagoya 467-8601, Japan

**Keywords:** breast cancer, radiotherapy, radiosensitization, hydrogen peroxide, hyperthermia, particle therapy, radiofrequency ablation, high-intensity focused ultrasound, cryoablation, microwave ablation

## Abstract

**Simple Summary:**

For breast cancer patients who do not wish to undergo any form of surgery, various non-surgical treatments have been investigated. Radiotherapy is considered the most important modality, but conventional radiotherapy alone or concurrent chemoradiotherapy do not lead to high local control rates. So, to increase curability, radiosensitization strategies, including the use of hydrogen peroxide injection before radiation and hyperthermia plus oral tegafur-gimeracil-oteracil potassium (S-1), have been investigated. These strategies have yielded promising outcomes, with local control rates of ~97%. A trial of carbon ion radiotherapy is ongoing, and particle therapy should be further investigated in the future. Image-guided ablation therapy including radiofrequency ablation and focused ultrasound have been investigated; although complete ablation rates of ≥70% have been reported, combination with radiotherapy may be necessary to treat the extensive intraductal components. Non-surgical treatment of breast cancer is evolving steadily and will become a valuable treatment option for patients who refuse surgery.

**Abstract:**

This article reviews the results of various non-surgical curative treatments for operable breast cancer. Radiotherapy is considered the most important among such treatments, but conventional radiotherapy alone and concurrent chemoradiotherapy do not achieve high cure rates. As a radiosensitization strategy, intratumoral injection of hydrogen peroxide before radiation has been investigated, and high local control rates (75–97%) were reported. The authors treated 45 patients with whole-breast radiotherapy, followed by stereotactic or intensity-modulated radiotherapy boost, with or without a radiosensitization strategy employing either hydrogen peroxide injection or hyperthermia plus oral tegafur-gimeracil-oteracil potassium. Stages were 0–I in 23 patients, II in 19, and III in 3. Clinical and cosmetic outcomes were good, with 5-year overall, progression-free, and local recurrence-free survival rates of 97, 86, and 88%, respectively. Trials of carbon ion radiotherapy are ongoing, with promising interim results. Radiofrequency ablation, focused ultrasound, and other image-guided ablation treatments yielded complete ablation rates of 20–100% (mostly ≥70%), but long-term cure rates remain unclear. In these treatments, combination with radiotherapy seems necessary to treat the extensive intraductal components. Non-surgical treatment of breast cancer is evolving steadily, with radiotherapy playing a major role. In the future, proton therapy with the ultra-high-dose-rate FLASH mode is expected to further improve outcomes.

## 1. Introduction

Breast cancer is increasing worldwide; there were over 2.3 million new cases and 685,000 deaths from breast cancer in 2020 in the world, but by 2040, new cases will increase to over 3 million, and 1 million patients will die every year [1]. Therefore, the choice of an optimal treatment for each patient, taking the patient’s wishes into consideration, may become more important in the future. Breast cancers are classified into subtypes based on the hormone-receptor positivity, human epidermal growth factor receptor type 2 (HER2) status, and proliferative activity of the tumor cells, and they are staged according to the size and local invasiveness of the tumor and the nodal and distant metastasis statuses [2]. For each subtype and stage, recommended neoadjuvant and adjuvant treatments exist, but the major treatment modality is surgery in the vast majority of non-Stage IV patients, unless they are medically inoperable or very elderly.

In fact, 95.7% of non-Stage IV breast cancer patients received surgery, according to a US National Cancer Database analysis [3]. Depending upon the disease stage and local status, breast-conserving surgery, with or without postoperative radiation or total mastectomy, is chosen. After breast-conserving surgery, however, the breast shape is not the same as before, and permanent scars remain, sometimes causing pain. Recently, prosthetic breast reconstruction was developed, but the reconstructed breast is an artificial one. In some patients, the mental burden and fear of undergoing surgery seem to be substantial. Indeed, the breasts are important body parts for women of any age, and so, if breast cancer can be cured without any surgical procedure, many women may wish to choose the non-surgical treatment. It may be natural that women wish to conserve their breasts as they are, and the progress in medicine and technology should meet the wishes of such women.

Non-surgical treatments of Stage 0-III operable breast cancer have been attempted in a relatively small proportion of patients who definitely refuse surgery. With the developments of imaging modalities, non-surgical treatment may now be delivered more precisely than before [4,5]. Radiation therapy has been used in inoperable cases, so it could be employed for definitive treatment. The National Cancer Database study emphasized the role of radiation therapy [3]. To achieve sufficient local control rates, however, some strategies to increase the radiation doses from conventionally used ones or to enhance the effects of radiation are necessary. In this respect, certain advances have been noted in recent years. The authors have attempted to treat patients who refuse surgery with radiation therapy, followed by stereotactic or intensity-modulated radiotherapy (IMRT) boost, with or without radiosensitization methods [6,7], and the number of patients so treated is increasing. Besides radiation therapy, image-guided ablative therapy has been attempted; there are various methods for this purpose, including radiofrequency ablation (RFA), high-intensity focused ultrasound (HIFU), cryoablation, and microwave ablation. Clinical data on these treatments are accumulating. In this article, the outcomes of these non-surgical treatments are reviewed, and the authors’ updated data are presented. Then, the future prospects of these treatments are discussed. A literature search was conducted in PubMed using various combinations of key words, such as breast cancer, radiotherapy, radiosensitization, hydrogen peroxide, hyperthermia, particle therapy, radiofrequency ablation, high-intensity focused ultrasound, cryoablation, and microwave ablation.

## 2. Radiotherapy-Based Treatment

### 2.1. Conventional Radiotherapy Alone

Radiation therapy has been used to treat unresectable breast cancer or elderly patients. Conventional fractionation or hypofractionation with conventional doses was used, so such treatments were not definitive ones. Previous data indicated that with conventional radiation doses, 3-year local control rates would be expected to be 45–57% [8,9,10]; these rates are insufficient as a definitive treatment. In a relatively large retrospective analysis of 192 patients with locally advanced breast cancer, patients were treated with 45–50 Gy to the breast, and about 80% of the patients received a local boost. Furthermore, 28% of the patients received multi-agent chemotherapy. As a result, however, the 5-year local control rate was 73%, and 5-year survival was 41% [11]. In another study of early breast cancer patients treated by primary radiotherapy, 27 tumors were found to have histologic features of moderate to marked intraductal carcinoma in the tumor and adjacent tissue and a high nuclear grade; the 5-year local control rate was 84% for 15 patients receiving ≥60 Gy, whereas it was 48% for those who received <60 Gy [12]. Hypofractionated treatment was used for more palliative cases [13], so data from such treatment are not fully evaluable. Combination with adjuvant hormone therapy would improve the survival time of hormone-receptor-positive patients [13].

### 2.2. Concurrent Chemoradiotherapy

Since conventional radiotherapy alone is insufficient to achieve a high local control rate, concurrent chemoradiotherapy has been investigated for early breast cancer by a few groups [14,15,16]. In a case series of 5 patients, 4 achieved local control for more than 2.5 years, but 1 developed local recurrence, which was treated by reirradiation [14]. The Japanese Clinical Oncology Group investigated preoperative chemoradiation for 108 Stage I–IIIA patients. All patients underwent mastectomy or lumpectomy thereafter, and a pathological complete response was achieved in only 36% of the patients [15]. Therefore, the conclusion of the study was that the treatment was not sufficient to use as a definitive treatment. In locally advanced breast cancer patients, concurrent chemoradiation was investigated by many groups in a preoperative neoadjuvant setting [16,17,18]; surgery was a prerequisite of treatment, and cure was not a goal of chemoradiation. Histological evaluation of resected specimens showed pathological complete response rates of 9–61% (median, 29%) [16]. Concurrent chemoradiotherapy in combination with neoadjuvant/adjuvant chemotherapy and hormonal therapy when indicated should be a method of definitive treatment, but intensification of radiation therapy may be necessary to achieve a high enough local control rate.

### 2.3. Radiotherapy with Hydrogen Peroxide Sensitization

As a definitive treatment for early breast cancer, Ogawa et al. [19] developed a new treatment modality named KORTUC (Kochi Oxydol-Radiation Therapy for Unresectable Carcinomas). In this treatment, hydrogen peroxide was injected into the breast tumor just before radiation therapy [20,21]. Hydrogen peroxide produces oxygen in the tumor and, hence, sensitizes hypoxic tumor cells to radiotherapy [22]. In addition, hydrogen peroxide inactivates anti-oxidative enzymes, such as peroxidases and catalases that are scavengers of radicals produced by radiation and reduce the therapeutic efficacy of RT [19]. Usually, hydrogen peroxide dissolved in sodium hyaluronate was injected twice a week, while radiation was delivered five times a week. Radiation doses used by Ogawa’s group were 44 Gy in 16 fractions (2.75 Gy per day) to the whole breast, followed by an electron boost with 9 Gy in 3 fractions [20,21]. They treated 72 patients with Stage I or II operable breast cancer with KORTUC, with or without chemotherapy and hormonal therapy. During a mean follow-up period of 51 months, they found only 1 local recurrence; another patient developed bone metastases. Disease-free survival and local control rates were both 97.1% at 5 years.

Following the study of Ogawa et al. [19,20,21], several groups used the intratumoral hydrogen peroxide radiosensitization method to treat breast cancer [6,7,8,23,24]. Table 1 summarizes the results reported so far. Subsequent investigators treated more advanced cases, and the treatment outcomes were not as good as those of Ogawa et al. Shimbo et al. [23] treated 30 patients with locally advanced or recurrent breast cancer employing the KORTUC method, and the 3-year local control rate was 75%; the 2-year progression-free survival rate was only 24%. Obata et al. [24] treated 5 patients with Stage I breast cancer with KORTUC, and no local recurrences or distant metastases have been observed during a median follow-up period of 65 months (range, 47–91 months). They also treated 2 Stage II patients with axillary lymph node metastases: one developed a local recurrence at 12 months, and another developed brain metastasis at 35 months and died at 56 months (personal communication, December 2022). In addition, Obata et al. [24] treated 32 patients with Stage III or IV breast cancer, and a complete or partial response was obtained in 50%. So, the treatment efficacy depends on the disease stage. Our group also used this treatment, and the results are shown in the next section, as well as Table 1.

This radiosensitization method with intratumoral hydrogen peroxide injection has spread to the United Kingdom, and a Phase I study was conducted for locally advanced breast cancer [8]. Twelve patients were treated, and all had acceptable toxicity. At the last imaging assessment, the percentage of tumor volume reduction was between 50 and 100%. A Phase II study is now being conducted.

### 2.4. Whole-Breast Radiotherapy Followed by Stereotactic or Intensity-Modulated Boost with or without Radiosensitization Strategy

The authors’ group has been using conventional whole-breast radiation, followed by stereotactic or IMRT boost, for operable breast cancer patients who refuse any type of surgery. Details of the treatment have been described [6,7]; updated results are shown in this article. Until recently, we used 50 Gy in 25 daily fractions for whole-breast treatment, but currently moderate hypofractionation with 44.8 Gy in 16 fractions (2.8 Gy daily) is used. Standard boost doses were 21 Gy in 3 fractions for stereotactic irradiation and 20 Gy in 8 fractions for IMRT. The planning target volume for the boost treatment was the internal target volume plus 5 mm margins in all directions. The IMRT boost dose has recently been modified to 19.6 Gy in 7 fractions (2.8 Gy daily). For tumors approximately ≥2 cm in maximum diameter, two types of radiosensitization methods have been applied: one is hydrogen peroxide injection (KORTUC) during whole-breast radiotherapy, and the other is hyperthermia plus oral tegafur-gimeracil-oteracil potassium (S-1). The former radiosensitization method was terminated due to a change in medical legislation in Japan, and thereafter, the latter sensitization method has been employed. The hydrogen peroxide radiosensitization method is the same as that used by Ogawa et al. [19,20,21]. Radiofrequency hyperthermia was performed with RF-8 (Yamamoto Vinitor, Osaka, Japan) once a week during radiation therapy up to five times. The skin temperature was maintained at 40–41.5 °C for at least 30 min. S-1 (80–120 mg/day) was orally administered twice a day from the evening before the starting day (usually Monday) of irradiation to the morning of weekends (usually Friday) and repeated until the treatment end.

**Figure 1:**
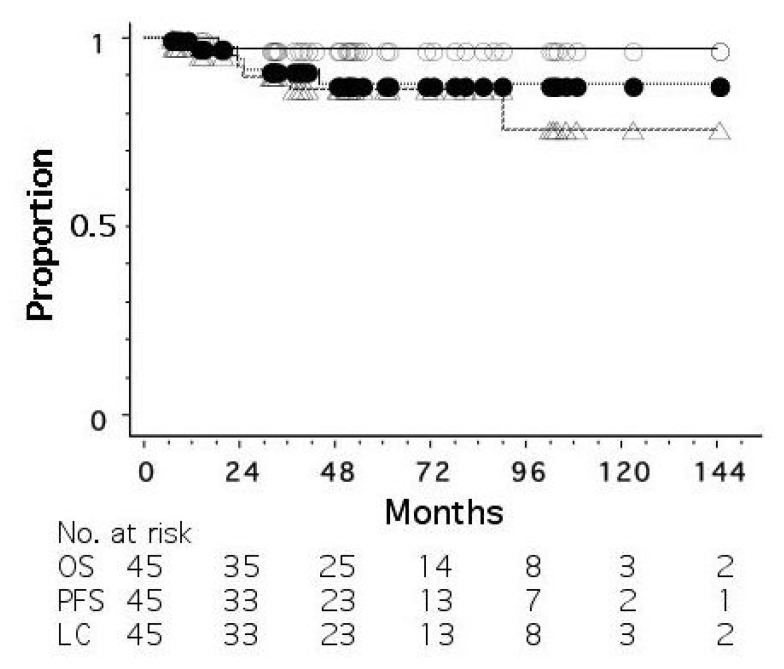
Overall (○), progression-free (△), and local recurrence-free (●) survival curves for 45 patients with operable breast cancer treated by whole-breast radiotherapy and stereotactic or intensity-modulated boost.

**Figure 2:**
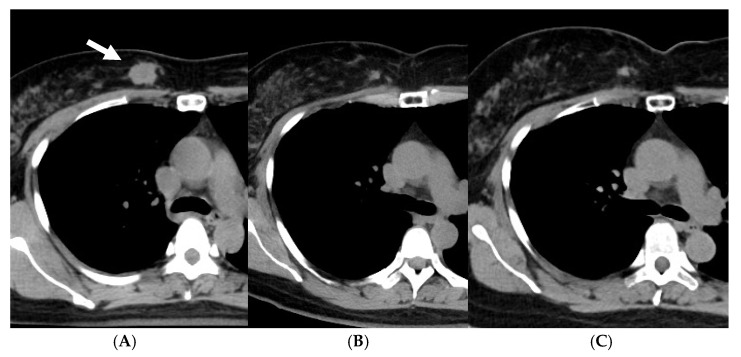
A case with an invasive ductal carcinoma (arrow) and a residual mass after treatment with whole-breast radiotherapy and stereotactic boost. (**A**) Before, (**B**) 22 months after, and (**C**) 81 months after treatment.

As of June 2022, 45 patients had been treated. The disease stages were 0 (ductal carcinoma in situ, DCIS) in 7 patients, I in 16, II in 19, and III in 3. The patients with a biopsy result of DCIS were staged as 0, but it was unknown whether the biopsy result represented the whole tumor. All the patients were judged to be operable by breast surgeons. Standard chemotherapy and/or hormonal therapy was used when the patients agreed to receive them; 4 patients received systemic chemotherapy and/or anti-HER2 therapy, and 31 of 35 hormone-receptor-positive patients received adjuvant hormonal therapy. Figure 1 shows overall, progression-free, and local recurrence-free survival curves for all 45 patients. The median follow-up period was 50 months (range, 6–180). The 5-year overall, progression-free, and local recurrence-free survival rates were 97.3, 86.4, and 87.9%, respectively. An important finding in our study is that even when a residual mass persisted after radiotherapy, the mass did not necessarily show regrowth. Figure 2 shows the changes of breast cancer over time. The original tumor achieved a partial response, but has remained stable after 22 months since treatment. We consider that these residual masses are usually scars, while fibroadenoma was detected by biopsy after 3 years of this treatment in a patient. Overall, 24 of the 45 patients had such a residual mass, but 20 of them have not developed local recurrence.

**Figure 3:**
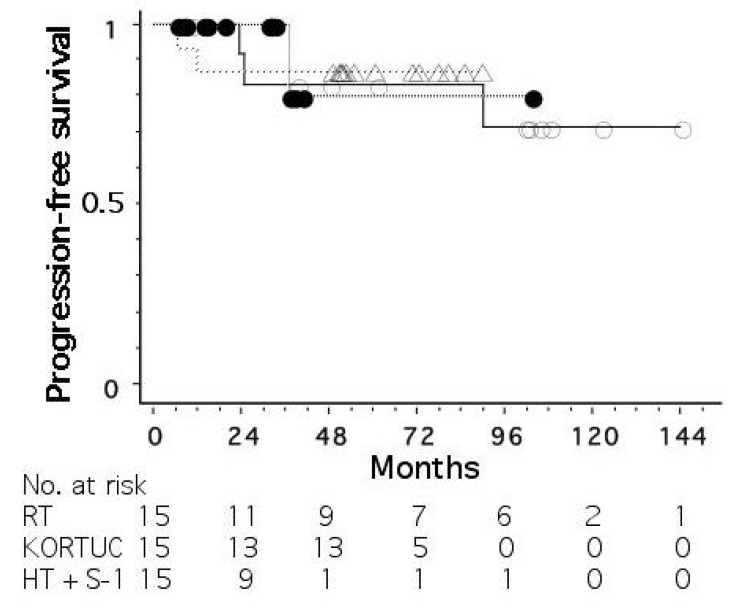
Progression-free survival curves for operable breast cancer patients treated with radiotherapy alone (○), radiotherapy plus hydrogen peroxide radiosensitization (△), and radiotherapy plus hyperthermia and oral tegafur-gimeracil-oteracil potassium (S-1) (●).

Figure 3 shows progression-free survival curves for the three groups treated without radiosensitization, with hydrogen peroxide sensitization, and with hyperthermia plus S-1 sensitization. The mean tumor size (±standard deviation) was 18 ± 11, 26 ± 9, and 27 ± 11 mm for the three groups, respectively. The stage distribution (0/I/II/III) was 2/9/3/1, 2/2/10/1, and 3/5/6/1, respectively. The 5-year progression-free survival rates were 83, 87, and 80%, respectively, for the three groups, with no significant differences among the groups.

Major toxicities were acute skin toxicities (radiation dermatitis), with Grade 1 in 23, Grade 2 in 7, and Grade 3 in 15. The Harvard Scale of breast cosmesis was excellent (nearly identical to an untreated breast) in 20, good (slightly different from an untreated breast) in 24, and fair (clearly different from an untreated breast but not seriously distorted) in 1. We reported enlargement of the irradiated breast probably due to lymph edema; although the enlarged breast is esthetically favorable, this produced asymmetry of the breasts, making the Harvard Scale “good” instead of “excellent”.

### 2.5. Particle Therapy

The use of proton or carbon ion beams has been considered for postoperative radiotherapy of breast cancer or for locally advanced cases [25,26]. Using proton beams in an anterior direction, irradiation to the lung can be minimized, and so it is expected that radiation pneumonitis would become almost zero. Attempts to use protons for definitive treatment of operable breast cancer are ongoing at two Japanese facilities of proton therapy. To our knowledge, however, patient accrual is limited, and no meaningful data are available at present.

Prospective studies of carbon ion therapy for Stage I breast cancer have been conducted. Results of a Phase I study, in which seven patients were enrolled, were reported [27]. They received hypofractionated carbon ion therapy with 48, 52.8, or 60 GyRBE in 4 fractions. At 3 months after carbon ion therapy, 1 achieved a complete response, 5 achieved a partial response, and 1 had stable disease. The tumors were excised at 3 months after the treatment and were histologically evaluated; among the 7 patients, only 2 had a Grade 3 pathological effect. The conclusion of the study was that the timing of the histological evaluation (at 3 months) may not have been optimal.

Subsequently, the study entered Phase II, and three studies have been conducted [28]. Although respective studies have limited patient numbers and are still ongoing, no local recurrence has been observed [28]. The most recent study employed a single fraction treatment with 42–50 GyRBE using partial-breast irradiation. In addition, the results for 14 off-protocol patients undergoing carbon ion therapy for Stage I (T1N0M0) breast cancer were reported [29]. Accelerated partial-breast irradiation was employed, and the radiation dose was 52.8 or 60 GyRBE in 4 fractions. Possibly due to the use of relatively high doses, 13 patients maintained a complete response, whereas only 1 patient developed local recurrence; this patient died of the disease at 69 months after carbon ion radiotherapy, while the other 13 patients were alive at 51–87 months (median, 61 months). Thus, particle therapy has not yet been established as a definitive treatment of early breast cancer, but carbon ion therapy may be worthy of further investigation.

## 3. Image-Guided Percutaneous Minimally Invasive Treatment

Percutaneous ablation therapy has been used for local treatment of benign breast tumors, and the indication has been extended to breast cancer. Ablation therapy includes RFA, HIFU, cryoablation, microwave ablation, and laser therapy. Vacuum-assisted excision and irreversible electroporation may become used in the future. Further, photothermal therapy and magnetic hyperthermia are under investigation. In most ablation treatments aiming at heat-induced coagulation necrosis, tumor temperatures are raised to >60 °C, in contrast to 40–42 °C in hyperthermia, in which a direct cell killing effect is considered to be weak, but a radiosensitizing effect is expected. In the ablation treatments, various types of cell death, including necrosis, apoptosis, ferroptosis, necroptosis, and pyroptosis, are reported to occur [30,31]. In hyperthermia, both necrosis and apoptosis were reported, and necrosis increased with elevation of the temperature above 45 °C [32]. In general, these modalities are applied to relatively small tumors (<2 cm), and breast cancers with extensive DCIS components are not indicated. Management of extensive intraductal components is always a problem in increasing the curability of these treatments, and in this respect, some groups employed radiation therapy in combination with ablation therapy. Below are brief summaries of each modality; for more details, readers are recommended to refer to other review articles [33,34,35,36,37,38,39,40,41,42,43].

### 3.1. Radiofrequency Ablation

RFA utilizes radiofrequency alternating current from electrodes placed in the tumor with the aid of ultrasound or magnetic resonance imaging (MRI). This current causes local coagulation necrosis. Cells usually die at temperatures > 60 °C, and shorter exposure times become sufficient as the temperature in the target increases. Regarding the procedure, RFA is advantageous in terms of a relatively short ablation time, use of a finer needle, no need for gas tanks, and the availability of MR thermometry. Disadvantages are a lack of monitoring the ablation zone during the procedure, the necessity of placing grounding pads on the patient, and the necessity of a tailored algorithm in some cases.

The clinical outcomes of representative studies are shown in Table 2, together with those of other modalities. In most studies of RFA, as well as other ablation treatments, ablated sites were excised later and underwent histopathological evaluation. The results showed a complete tumor eradication rate of 30–100% (mostly >60%) [37,44,45,46,47,48]. Studies without subsequent surgery are few, but they indicated local control rates of 70–100%; 100% local control was reported during a follow-up period of 6–30 months (median, 15 months), but a longer follow-up is clearly necessary [45]. Frequently observed complications of RFA are bleeding, infection, breast ecchymosis, skin burns, and fat necrosis.

### 3.2. High-Intensity Focused Ultrasound

In HIFU, high-frequency ultrasound waves (average spatial intensity, 100–10,000 W/cm^2^) are focused on the target under MRI or ultrasound guidance, and acoustic energy is converted to heat, producing thermal coagulation. A focal temperature of 56–90 °C for >1 s effectively produces thermal injury. Other proposed mechanisms for HIFU ablation are acoustic cavitation, microstreaming, and immune system modulation [57]. The advantages of HIFU are no probe insertion, leading to excellent cosmesis; sharp ablation margin; negligible cooling effect from blood flow; and inhomogeneous temperature distribution. The disadvantages are the relatively long treatment time (>40 min) and sensitivity to patient movement and near-field heating.

Similar to other ablation modalities, HIFU has been used before surgery to histologically evaluate its effect. Various studies reported complete necrosis rates of 17–100% (mostly >50%), which may be similar to those of other ablation modalities [33,37,39,49,58]. In a study with 21 patients followed by MRI after HIFU-alone treatment, only one recurrence was observed during a median follow-up period of 14 months (range: 3–26) [50]. In another study, HIFU was combined with radiation, chemotherapy, and hormonal therapy, and surgery was not performed; of 22 stage I–IV patients so treated, 2 developed local recurrence [51]. The 5-year disease-free and recurrence-free survival were 95 and 89%, respectively. Therefore, combination with radiochemotherapy and hormone treatment may yield better outcomes. Complications of HIFU include pain, edema, muscle injury, erythema, hyperpigmentation, and hemorrhage.

### 3.3. Cryoablation

Under ultrasound guidance, a needle is placed in the lesion, and an ice ball is expanded. The procedure consists of the initial freeze, passive thaw, and subsequent freeze. During the first freeze, extracellular water freezes earlier due to a higher intracellular osmolality, leading to dehydration of the cells. In the passive thaw phase, the osmotic gradient reverses, causing cell swelling and rupture. A longer thaw after the first freeze seems more effective than rapid freezing to increase cellular damage, since intracellular ice crystals growing while thawing disrupt organelles and plasma membranes [59]. The second freeze enlarges the necrotic area, since the tissue disrupted during the first freeze conducts cold temperature more efficiently. Tissue destruction occurs at lethal temperatures < −40 °C. The advantages of cryoablation are the good visualization and rapid procedure and recovery. The disadvantages are the cost and cumbersome management of argon.

In clinical studies of cryoablation in which treatment success was evaluated histopathologically, clinical success was reported in 18–99% (mostly >70%) of the patients [33,37,52,53]. However, studies without subsequent surgery have not been documented to date. Complications include skin frostbite, bleeding, infection, and skin or chest wall injury.

### 3.4. Microwave Ablation

Microwave ablation also creates frictional heat and induces coagulative necrosis. In contrast to RFA that uses resistive heating, microwave ablation heats tissue surrounding an antenna that transfers energy from a power source to tissue. At frequencies of 900–2500 MHz, the temperature reaches a lethal level. Since microwave ablation is less effective in low-water tissue like fat, tumor cells are considered to be more effectively destroyed than normal fatty breast tissue [60]. The delivery of energy in microwave ablation is less limited by the electrical impedance of tissue when compared to RFA, and so it theoretically increases the probability of complete ablation [61]. This is an advantage of microwave ablation. As a disadvantage, the procedure can be very painful and may be poorly tolerated by some patients.

A clinical study reported a complete tumor coagulation rate of 90% in 41 patients [54]. Imaging follow-up of the patients revealed tumor volume reduction over time, but the long-term outcomes of patients treated without subsequent surgery have not been reported. The complications of microwave ablation are mostly skin burns.

### 3.5. Laser Ablation

Laser therapy aims at elective thermal destruction of the target by converting light into thermal energy, causing direct and indirect damage to the tissue. Heat injury occurs directly during heat deposition, and indirect injury develops thereafter, producing progressive tissue damage from tissue vaporization, microvascular damage, tissue necrosis, and immune cell activation [55]. Under ultrasound or stereotactic guidance, a laser fiber in a laser probe is placed in the lesion. A continuous-wave 805 nm diode laser and a neodymium-doped yttrium aluminum garnet (Nd:YAG) 1064 nm laser are most frequently employed. At the laser–tissue interface, the tissue absorbs photons and causes excitation and the release of thermal energy, which results in rapid temperature elevation and irreversible tissue damage. At temperatures of 60 °C, coagulation necrosis occurs rapidly, while supraphysiologic hyperthermia is delivered to surrounding tissues, resulting in delayed thermal damage. Overheating may reduce its effectiveness, since tissue carbonization may alter tissue optical properties and hamper penetration. In this modality, major complications are rare. As disadvantages, carbonization may limit penetration, and MRI is required to evaluate the ablation zone. In addition, the treatment parameters need to be adapted for patient-specific tissue.

In clinical studies, complete tumor ablation was reported in 84% of 61 patients with early breast cancer [55] and 70% of 54 patients with breast cancer smaller than 1.5 cm [56]. In the latter study, 2 patients were followed for up to 24 months without resection; tumor shrinkage and subsequent oil cyst formation and fibrosis at biopsy were reported. The complications of laser ablation are skin burns, hyperpigmentation, and pneumothorax.

### 3.6. Other Modalities

Two other ablation methods may be considered for future application in the treatment of early breast cancer [33]. An ultrasound-guided vacuum-assisted breast biopsy device comprising a cutting needle and vacuum suction can be used for the treatment of small breast lesions, such as fibroadenoma; however, to date, vacuum-assisted excision has not yet been used for breast cancer. Excising the tumor from multiple directions and repeating the excision cycle may be required for complete resection.

In irreversible electroporation, electric pulses are delivered at high voltages through needle electrodes placed around the tumor. Brief intense electric pulses alter the membrane potential and produce permanent nanopores in the cell membrane, increasing its permeability. Irreversible apoptosis also occurs above a certain threshold. Then, a well-demarcated ablation zone is produced with a sharp boundary. To date, this modality has also not been employed for the treatment of breast cancer.

Furthermore, two novel modalities are under investigation before clinical use. Photo-thermal therapy involves photo-thermoconversion agents and laser irradiation. Gold nanoparticles generate heat when exposed to light, especially in the near-infrared range. Compounds possessing various unique properties other than thermoconversion are being developed [62,63]. In magnetic hyperthermia, magnetic nanoparticles are injected into the tumor, and alternating the magnetic field induces heat by interacting with the nanoparticles [64,65]. The temperature reaches 43 °C or higher, leading to the apoptosis of cancer cells. Since the magnetic field is only absorbed by the magnetic nanoparticles, cancer cells can be treated selectively. These two methods have not yet been investigated as clinical trials, but may deserve further investigation as new treatment modalities for breast cancer.

### 3.7. Summary of Ablation Therapies

All ablation therapy modalities may be effective in achieving tumor necrosis. Reported complete necrosis rates differ slightly with the modalities, but this may not be inherent to the differences in modalities. The complete ablation rate depends on the tumor size, nature of the breast cancer, patient selection criteria, imaging techniques used, ablation protocols, size of the ablated margins, and evaluation method. Therefore, the modality that is most frequently used in each institution may be employed. However, the greatest issue in using ablation therapy in the definitive treatment of operable breast cancers is the treatment of extensive intraductal components. To treat such components, whole-breast radiation therapy or partial-breast irradiation with adequate margins may be the most useful and effective, so the authors consider that combination with radiotherapy should be mandatory to establish the treatment as the definitive breast cancer therapy.

## 4. Current Recommendations and Future Prospects

From the above considerations, the use of whole-breast radiation is strongly recommended to cure operable breast cancer. When indicated, accelerated partial-breast irradiation may be employed in place of whole-breast irradiation. A tumor mass may be treated with a stereotactic or IMRT boost, as our group is doing, or with an ablation modality. Since skin reactions occur with whole-breast radiation, an ablation treatment may be better performed before whole-breast radiation. A stereotactic or IMRT boost should be given after whole-breast radiation, because the residual tumor can be treated with smaller radiation fields. It is not yet known which of ablation therapy and stereotactic or IMRT boost is better, and this should be determined in future studies. The advantage of IMRT boost is that multiple tumors can be readily treated in one treatment session. In addition, stereotactic radiation and IMRT boost may have an advantage regarding the treatment of tumor margins. In our study, the margin for the boost were 5 mm, which was not much different from the ablation margins (3–10 mm) in most studies [5,33,44]. However, while the effect of ablation treatment sharply decreases outside the margins, radiation doses fall off by gradation outside the margins, so a geographic miss may be less likely to occur in the radiation boost method. The disadvantage of stereotactic radiotherapy or IMRT boost is the skin toxicity, as we observed many patients with Grade 3 radiation dermatitis. However, the dermatitis later recovered to acceptable levels. Conventional electron boost may be sufficient to treat very small tumors (<1 cm), and for larger tumors, it should be employed when strategies to intensify the effect of whole-breast radiation, such as hydrogen peroxide injection, are used. Which of the radiosensitization strategies is the best is still unknown and should be determined in future studies. Besides radiosensitization efficacy, complications, especially with regard to cosmetic outcomes, should be evaluated. Hydrogen peroxide injection causes indurations of the injected site, and hyperthermia often leads to fat necrosis. It is desirable to avoid these complications.

The recommended treatment differs with the subtype of breast cancer. Of course, standard adjuvant chemotherapy and hormonal therapy should be used whenever indicated to increase the curability. HER2-type breast cancer responds to anti-HER2 therapy, and a pathological complete response is often obtained. In such cases, simply adding whole- or partial-breast irradiation may be sufficient to cure the tumor. A prospective study to test this hypothesis is ongoing by the Japan Clinical Oncology Group (JCOG) Breast Cancer Study Group (JCOG1806: http://www.jcog.jp/basic/org/group/bcsq.html, accessed on 25 January 2023). This approach should be investigated in future studies.

Whole-breast irradiation is usually delivered with photons using tangential fields, by which irradiation to the lung can be kept at a low level. However, Grade I radiation pneumonitis develops in most patients in the lung adjacent to the breast. IMRT can reduce the dose to the lung, but it does not become zero. In addition, irradiation to the heart becomes an issue in left-sided breast cancer, although irradiation during deep breathing can decrease radiation doses to the heart. A good solution to this issue is the use of particle therapy, especially proton therapy. When protons are irradiated from the anterior direction, the dose to the lung could be minimal among various radiation methods. When the cost–benefit issue is solved, proton beam therapy may become the best choice in the future. Further, when using accelerated partial-breast irradiation, proton or carbon ion radiotherapy may be effective.

Recently, ultra-high-dose-rate (>40 Gy/s) FLASH radiotherapy has been attracting marked attention among various research topics in radiation oncology [66,67,68]. In terms of the mechanisms of producing the high-dose rate, electron and particle beams seem to be more suitable to produce the ultra-high-dose rate than photon beams. In the future, FLASH electron beams may be employed for a boost after whole-breast radiotherapy for superficial breast cancer. Since one of the proposed mechanisms for the FLASH effect is oxygen depletion in normal tissue [69], proton beams may produce stronger FLASH effects than heavy ions, because the oxygen effect (oxygen enhancement ratio) is greater for protons than heavy ions [70]. So, to treat breast cancers, including relatively deep-seated ones, proton beams may be the best. To obtain the normal tissue-sparing effects of FLASH therapy, a high fractional dose is required, so FLASH proton beams may be used for the boost treatment after whole-breast radiotherapy. In a mouse model, radioprotective effects for the skin has been reported with a dose-modifying factor of 1.44–1.58 [71]. Skin reactions are marked at the completion of whole-breast radiotherapy, so the use of FLASH protons may be a better choice for patients without lymph node metastasis. Thus, radiation therapy is expected to further develop in the future as a key treatment modality for operable breast cancer patients who do not wish to undergo surgery.

## 5. Conclusions

Non-surgical definitive treatment is gradually being developed for early breast cancer and will be established in the near future. This should be good news for patients who do not wish to receive any surgery. With the increase in treatment options, computational modeling and treatment planning may play a greater role in the future. Further investigations to determine the optimal treatment modality for this purpose are warranted.

## Figures and Tables

**Table 1:** Studies on radiosensitization with intratumoral hydrogen peroxide injection for breast cancer.

1st Author	*n*	Stage	Radiation (Gy/Fraction)	OS (%)	PFS (%)	LC (%)
Ogawa [20]	72	I/II	44/16 + 9/3	100 (5 y)	97.1 (5 y)	97.1 (5 y)
Shimbo [23]	30	IIIA–IV & R	44–67/16–30	60 (3 y)	24 (2 y)	75 (3 y)
Obata [24] *	5	I	44/16 + 9/3	100 (5 y)	100 (5 y)	100 (5 y)
Nimalasena [8]	13	I–IV	36/6 or 49.5/18	92 (1 y)	NA	100 (1 y)
Shibamoto [7] **	15	I–III	50/25 + 21/3 or 20/8	93 (5 y)	87 (5 y)	93 (5 y)

Abbreviations: OS = overall survival; PFS = progression-free survival; LC = local control; y = years; R = recurrence; NA = not available; CR = complete response. * Updated by personal communication. ** Updated in this study.

**Table 2:** Excerpts of clinical studies of ablation therapy for breast cancer.

1st Author	Modality	*n*	Size * (cm)	Other Therapy	Complete Necrosis Rate (%)	Local Control (%)	Survival (%)
Ito [44]	RFA	244	≤1.0	R (91%)	NA	97	NA
		111	1.1–2.0		NA	94	NA
		30	>2.0		NA	87	NA
Oura [45]	RFA	52	0.5–2.0 (1.3)	S, R	42	100	100 (6–30 M)
Kinoshita [46]	RFA	49	<3.0 (1.7)	S	61	NA	NA
Yamamoto [47]	RFA	29	0.5–1.9 (1.3)	S, C, H	92	NA	NA
Palussière [48]	RFA	21	<3.0	H, S (-)		95	NA
Gianfelice [49]	HIFU	24	<2.0	H, S (-)	79	NA	NA
Furusawa [50]	HIFU	21	0.5–5.0 (1.5)	R, C		95 (~26 M)	NA
Wu [51]	HIFU	22	2.0–4.8 (3.4)	R, C, H		89 (5 year)	NA
Simmons [52]	Cryo	87	0–1.9 (1.1)	S	76	NA	NA
Poplack [53]	Cryo	20	≤1.5	S	85	NA	NA
Zhou [54]	Microwave	41	1.3–6.4 (2.2)	S	90	NA	NA
Schwartzberg [55]	Laser	61	0.4–1.9 (1.1)	S, R, C	84	96 (34–65 M)	NA
Dowlatshahi [56]	Laser	54	0.5–2.3 (1.2)	S	70	NA	NA

* Data in parentheses are mean. Abbreviations: RFA = radiofrequency ablation; NA = not available; S = surgery; (-) = not performed; R = radiation; M = months; C = chemotherapy; H = hormone therapy; HIFU = high-intensity focused ultrasound; Cryo = cryosurgery.

## Data Availability

Data generated or analyzed during the study are available from the corresponding author by request.

## References

[1] Arnold M., Morgan E., Rumgay H., Mafra A., Singh D., Laversanne M., Vignat J., Gralow J.R., Cardoso F., Siesling S. (2022). Current and future burden of breast cancer: Global statistics for 2020 and 2040. Breast.

[2] Waks A.G., Winer E.P. (2019). Breast cancer treatment: A review. JAMA.

[3] Boyce-Fappiano D., Bedrosian I., Shen Y., Lin H., Gjyshi O., Yoder A., Shaitelman S.F., Woodward W.A. (2021). Evaluation of overall survival and barriers to surgery for patients with breast cancer treated without surgery: A National Cancer Database analysis. NPJ Breast Cancer.

[4] Singh M., Dalal M., Sodhi G.S. (2021). Estimation of Clinical Size of Breast Tumour Lesions Using Contrast Enhanced Magnetic Resonance Imaging: Delineation of Tumour Boundaries.

[5] Singh M., Singh T., Soni S. (2020). Pre-operative assessment of ablation margins for variable blood perfusion metrics in a magnetic resonance imaging based complex breast tumour anatomy: Simulation paradigms in thermal therapies. Comput. Methods Programs Biomed..

[6] Shibamoto Y., Murai T., Suzuki K., Hashizume C., Ohta K., Yamada Y., Niwa M., Torii A., Shimohira M. (2018). Definitive radiotherapy with SBRT or IMRT boost for breast cancer: Excellent local control and cosmetic outcome. Technol. Cancer Res. Treat..

[7] Shibamoto Y., Takano S., Iida M., Urano M., Ohta K., Oguri M., Murai T. (2022). Definitive radiotherapy with stereotactic or IMRT boost with or without radiosensitization strategy for operable breast cancer patients who refuse surgery. J. Radiat. Res..

[8] Nimalasena S., Gothard L., Anbalagan S., Allen S., Sinnett V., Mohammed K., Kothari G., Musallam A., Lucy C., Yu S. (2020). Intratumoral hydrogen peroxide with radiation therapy in locally advanced breast cancer: Results from a Phase 1 clinical trial. Int. J. Radiat. Oncol. Biol. Phys..

[9] Arriagada R., Mouriesse H., Sarrazin D., Clark R.M., Deboer G. (1985). Radiotherapy alone in breast cancer. I. Analysis of tumor parameters, tumor dose and local control: The experience of the Gustave-Roussy Institute and the Princess Margaret Hospital. Int. J. Radiat. Oncol. Biol. Phys..

[10] Bedwinek J., Rao D.V., Perez C., Lee J., Fineberg B. (1982). Stage III and localized stage IV breast cancer: Irradiation alone vs irradiation plus surgery. Int. J. Radiat. Oncol. Biol. Phys..

[11] Sheldon T., Hayes D.F., Cady B., Parker L., Osteen R., Silver B., Recht A., Come S., Henderson I.C., Harris J.R. (1987). Primary radiation therapy for locally advanced breast cancer. Cancer.

[12] Harris J.R., Connolly J.L., Schnitt S.J., Cohen R.B., Hellman S. (1983). Clinical-pathologic study of early breast cancer treated by primary radiation therapy. J. Clin. Oncol..

[13] Courdi A., Ortholan C., Hannoun-Levi J.M., Ferrero J.M., Largillier R., Balu-Maestro C., Chapellier C., Ettore F., Birtwisle-Peyrottes I. (2006). Long-term results of hypofractionated radiotherapy and hormonal therapy without surgery for breast cancer in elderly patients. Radiother. Oncol..

[14] Kao P., Chi M.-S., Chi K.-H., Ko H.-L. (2019). Primary chemo-radiotherapy for breast cancer patients who refused surgical treatment: A case series. Ther. Radiol. Oncol..

[15] Mukai H., Watanabe T., Mitsumori M., Tsuda H., Nakamura S., Masuda N., Yamamoto N., Shibata T., Sato A., Iwata H. (2013). Final results of a safety and efficacy trial of preoperative sequential chemoradiation therapy for the nonsurgical treatment of early breast cancer: Japan Clinical Oncology Group Study JCOG0306. Oncology.

[16] Ciérvide R., Montero Á., García-Rico E., García-Aranda M., Herrero M., Skaarup J., Benassi L., Barrera M.J., Vega E., Rojas B. (2022). Primary chemoradiotherapy treatment (PCRT) for HER2+ and triple negative breast cancer patients: A feasible combination. Cancers.

[17] Shanta V., Swaminathan R., Rama R., Radhika R. (2008). Retrospective analysis of locally advanced noninflammatory breast cancer from Chennai, South India, 1990–1999. Int. J. Radiat. Oncol. Biol. Phys..

[18] Matuschek C., Boelke E., Roth S.L., Orth K., Lang I., Bojar H., Janni J.W., Audretsch W., Nestle-Kraemling C., Lammering G. (2012). Long-term outcome after neoadjuvant radiochemotherapy in locally advanced noninflammatory breast cancer and predictive factors for a pathologic complete remission. Results of a multivariate analysis. Strahlenther. Onkol..

[19] Ogawa Y., Kubota K., Ue H., Kataoka Y., Tadokoro M., Miyatake K., Tsuzuki K., Yamanishi T., Itoh S., Hitomi J. (2009). Phase I study of a new radiosensitizer containing hydrogen peroxide and sodium hyaluronate for topical tumor injection: A new enzyme-targeting radiosensitization treatment, Kochi Oxydol-Radiation Therapy for Unresectable Carcinomas, Type II (KORTUC II). Int. J. Oncol..

[20] Ogawa Y., Kubota K., Aoyama N., Yamanashi T., Kariya S., Hamada N., Nogami M., Nishioka A., Onogawa M., Miyamura M. (2015). Non-surgical breast-conserving treatment (KORTUC-BCT) using a new radiosensitization method (KORTUC II) for patients with Stage I or II breast cancer. Cancers.

[21] Aoyama N., Ogawa Y., Yasuoka M., Ohgi K., Iwasa H., Miyatake K., Yoshimatsu R., Yamanashi T., Hamada N., Tamura T. (2017). Therapeutic results of a novel enzyme-targeting radiosensitization treatment, Kochi oxydol-radiation therapy for unresectable carcinomas II, in patients with stage I primary breast cancer. Oncol. Lett..

[22] Takaoka T., Shibamoto Y., Matsuo M., Sugie C., Murai T., Ogawa Y., Miyakawa A., Manabe Y., Kondo T., Nakajima K. (2017). Biological effects of hydrogen peroxide administered intratumorally with or without irradiation in murine tumors. Cancer Sci..

[23] Shimbo T., Nakata M., Yoshioka H., Sato C., Hori A., Kimura K., Iwamoto M., Yoshida K., Uesugi Y., Akiyama H. (2021). New enzyme-targeting radiosensitizer (KORTUC II) treatment for locally advanced or recurrent breast cancer. Mol. Clin. Oncol..

[24] Obata S., Ishimaru Y., Miyagi S., Nakatake M., Kuroiwa A., Ohta Y., Kan T., Kanegae S., Inoue Y., Nishizato R. (2022). Actual practice of Kochi Oxydol Radiation Therapy for Unresectable Carcinomas by intra-tumoral administration of hydrogen peroxide as a radiosensitizer. Mol. Clin. Oncol..

[25] Roman O., Kowalchuk R.O., Corbin K.S., Jimenez R.B. (2022). Particle therapy for breast cancer. Cancers.

[26] Malouff T.D., Mahajan A., Mutter R.W., Krishnan S., Hoppe B.S., Beltran C., Trifiletti D.M., Vallow L.A. (2020). Carbon ion radiation therapy in breast cancer: A new frontier. Breast Cancer Res. Treat..

[27] Karasawa K., Omatsu T., Arakawa A., Yamamoto N., Ishikawa T., Saito M., Fukuda S., Kamada T., Working Group for Breast Cancer (2019). A Phase I clinical trial of carbon ion radiotherapy for Stage I breast cancer: Clinical and pathological evaluation. J. Radiat. Res..

[28] Karasawa K., Murata K., Mori Y., Okonogi N., Omatsu N., Wakatsuki M., Yamada S. Clinical trials of carbon-ion therapy for early breast cancer. Proceedings of the 35th Annual Meeting JASTRO.

[29] Karasawa K., Omatsu T., Shiba S., Irie D., Wakatsuki M., Fukuda S. (2020). A clinical study of curative partial breast irradiation for stage I breast cancer using carbon ion radiotherapy. Radiat. Oncol..

[30] Brock R.M., Beitel-White N., Davalos R.V., Allen I.C. (2020). Starting a fire without flame: The induction of cell death and inflammation in electroporation-based tumor ablation strategies. Front. Oncol..

[31] Yu L., Cheng M., Liu J., Ye X., Wei Z., Xu J., Xie Q., Liang J. (2023). Crosstalk between microwave ablation and ferroptosis: The next hot topic?. Front. Oncol..

[32] Zhou J., Wang X., Du L., Zhao L., Lei F., Ouyang W., Zhang Y., Liao Y., Tang J. (2011). Effect of hyperthermia on the apoptosis and proliferation of CaSki cells. Mol. Med. Rep..

[33] Roknsharifi S., Wattamwar K., Fishman M.D.C., Ward R.C., Ford K., Faintuch S., Joshi S., Dialani V. (2021). Image-guided microinvasive percutaneous treatment of breast lesions: Where do we stand?. Radiographics.

[34] Grotenhuis B.A., Vrijland W.W., Klem T.M.A.L. (2013). Radiofrequency ablation for early-stage breast cancer: Treatment outcomes and practical considerations. Eur. J. Surg. Oncol..

[35] Dai Y., Ping Liang P., Yu J. (2022). Percutaneous management of breast cancer: A systematic review. Curr. Oncol. Rep..

[36] van der Voort E.M.F., Struik G.M., Birnie E., Moelker A., Verhoef C., Klem T.M.A.L. (2021). Thermal ablation as an alternative for surgical resection of small (≤ 2 cm) breast cancers: A meta-analysis. Clin. Breast Cancer.

[37] Pediconi F., Marzocca F., Marincola B.C., Napoli A. (2018). MRI-guided treatment in the breast. J. Magn. Reson. Imaging.

[38] Xia L.Y., Hu Q.L., Xu W.Y. (2021). Efficacy and safety of radiofrequency ablation for breast cancer smaller than 2 cm: A systematic review and meta-analysis. Front. Oncol..

[39] Schmitz A.C., Gianfelice D., Daniel B.L., Mali W.P.T.M., van der Bosch M.A.A.J. (2008). Image-guided focused ultrasound ablation of breast cancer: Current status, challenges, and future directions. Eur. Radiol..

[40] Peek M.C.L., Ahmed M., Napoli A., ten Haken B., McWilliams S., Usiskin S.I., Pinder S.E., van Hemelrijck M., Douek M. (2015). Systematic review of high-intensity focused ultrasound ablation in the treatment of breast cancer. Br. J. Surg..

[41] Feril L.B., Fernan R.L., Tachibana K. (2021). High-intensity focused ultrasound in the treatment of breast cancer. Curr. Med. Chem..

[42] Takada M., Toi M. (2019). Cryosurgery for primary breast cancers, its biological impact, and clinical outcomes. Int. J. Clin. Oncol..

[43] Pusceddu C., Paliogiannis P., Nigri G., Fancellu A. (2019). Cryoablation in the management of breast cancer: Evidence to date. Breast Cancer.

[44] Ito T., Oura S., Nagamine S., Takahashi M., Yamamoto N., Yamamichi N., Earashi M., Doihara H., Imoto S., Mitsuyama S. (2018). Radiofrequency ablation of breast cancer: A retrospective study. Clin. Breast Cancer.

[45] Oura S., Tamaki T., Hirai I., Yoshimasu T., Ohta F., Nakamura R., Okamura Y. (2007). Radiofrequency ablation therapy in patients with breast cancers two centimeters or less in size. Breast Cancer.

[46] Kinoshita T., Iwamoto E., Tsuda H., Seki K. (2011). Radiofrequency ablation as local therapy for early breast carcinomas. Breast Cancer.

[47] Yamamoto N., Fujimoto H., Nakamura R., Arai M., Yoshii A., Kaji S., Itami M. (2011). Pilot study of radiofrequency ablation therapy without surgical excision for T1 breast cancer: Evaluation with MRI and vacuum-assisted core needle biopsy and safety management. Breast Cancer.

[48] Palussière J., Henriques C., Mauriac L., Asad-Syed M., Valentin F., Brouste V., Mathoulin-Pélissier S., de Lara C.T., Debled M. (2012). Radiofrequency ablation as a substitute for surgery in elderly patients with nonresected breast cancer: Pilot study with long-term outcomes. Radiology.

[49] Gianfelice D., Khiat A., Boulanger Y., Amara M., Belblidia A. (2003). Feasibility of magnetic resonance imaging-guided focused ultrasound surgery as an adjunct to tamoxifen therapy in high-risk surgical patients with breast carcinoma. J. Vasc. Intervent. Radiol..

[50] Furusawa H., Namba K., Nakahara H., Tanaka C., Yasuda Y., Hirabara E., Imahariyama M., Komaki K. (2007). The evolving non-surgical ablation of breast cancer: MR guided focused ultrasound (MRgFUS). Breast Cancer.

[51] Wu F., Wang Z.B., Zhu H., Chen W.Z., Zou J.Z., Bai J., Li K.Q., Jin C.B., Xie F.L., Su H.B. (2005). Extracorporeal high intensity focused ultrasound treatment for patients with breast cancer. Breast Cancer Res. Treat..

[52] Simmons R.M., Ballman K.V., Cox C., Carp N., Sabol J., Hwang R.F., Attai D., Sabel M., Nathanson D., Kenler A. (2016). A Phase II trial exploring the success of cryoablation therapy in the treatment of invasive breast carcinoma: Results from ACOSOG (Alliance) Z1072. Ann. Surg. Oncol..

[53] Poplack S.P., Levine G.M., Henry L., Wells W.A., Heinemann F.S., Hanna C.M., Deneen D.R., Tosteson T.D., Barth R.J. (2015). A pilot study of ultrasound-guided cryoablation of invasive ductal carcinomas up to 15 mm with MRI follow-up and subsequent surgical resection. Am. J. Roentgenol..

[54] Zhou W., Zha X., Liu X., Ding Q., Chen L., Ni Y., Zhang Y., Xu Y., Chen L., Zhao Y. (2012). US-guided percutaneous microwave coagulation of small breast cancers: A clinical study. Radiology.

[55] Schwartzberg B., Lewin J., Abdelatif O., Bernard J., Bu-Ali H., Cawthorn S., Chen-Seetoo M., Feldman S., Govindarajulu S., Jones L. (2018). Phase 2 open-label trial investigating percutaneous laser ablation for treatment of early-stage breast cancer: MRI, pathology, and outcome correlations. Ann. Surg. Oncol..

[56] Dowlatshahi K., Francescatti D.S., Bloom K.J. (2002). Laser therapy for small breast cancers. Am. J. Surg..

[57] Copelan A., Hartman J., Chehab M., Venkatesan A.M. (2015). High-intensity focused ultrasound: Current status for image-guided therapy. Semin. Intervent. Radiol..

[58] Furusawa H., Namba K., Thomsen S., Akiyama F., Bendet A., Tanaka C., Yasuda Y., Nakahara H. (2006). Magnetic resonance-guided focused ultrasound surgery of breast cancer: Reliability and effectiveness. J. Am. Coll. Surg..

[59] Baust J.G., Gage A.A. (2005). The molecular basis of cryosurgery. BJU Int..

[60] Roubidoux M.A., Yang W., Stafford R.J. (2014). Image-guided ablation in breast cancer treatment. Tech. Vasc. Interv. Radiol..

[61] Yang Q., Li H., Chen B.H., He G.Z., Wu X.P., Wang L.X., Wu H., Dou J.P., Han Z.Y., Zhang J. (2020). Ultrasound-guided percutaneous microwave ablation for 755 benign breast lesions: A prospective multicenter study. Eur. Radiol..

[62] Ayala-Orozco C., Urban C., Bishnoi S., Urban A., Charron H., Mitchell T., Shea M., Nanda S., Schiff R., Halas N. (2014). Sub-100 nm gold nanomatryoshkas improve photo-thermal therapy efficacy in large and highly aggressive triple negative breast tumors. J. Control Release.

[63] Rahimi-Moghaddam F., Sattarahmady N., Azarpira N. (2019). Gold-curcumin nanostructure in photo-thermal therapy on breast cancer cell line: 650 and 808 nm diode lasers as light sources. J. Biomed. Phys. Eng..

[64] Cao T.L., Le T.A., Hadadian Y., Yoon J. (2021). Theoretical analysis for using pulsed heating power in magnetic hypertheremia therapy of breast cancer. Int. J. Mol. Sci..

[65] Rahpeima R., Lin C.A. (2022). Numerical study of magnetic hyperthermia ablation of breast tumor on an anatomically realistic breast phantom. PLoS ONE.

[66] Favaudon V., Caplier L., Monceau V., Pouzoulet F., Sayarath M., Fouillade C., Poupon M.F., Brito I., Hupé P., Bourhis J. (2014). Ultrahigh dose-rate FLASH irradiation increases the differential response between normal and tumor tissue in mice. Sci. Transl. Med..

[67] Vozenin M.C., Hendry J.H., Limoli C.L. (2019). Biological benefits of ultra-high dose rate FLASH radiotherapy: Sleeping beauty awoken. Clin. Oncol..

[68] Wilson J.D., Hammond E.M., Higgins G.S., Petersson K. (2020). Ultra-high dose rate (FLASH) radiotherapy: Silver bullet or fool’s gold?. Front. Oncol..

[69] Kusumoto T., Inaniwa T., Mizushima K., Sato S., Hojo S., Kitamura H., Konishi T., Kodaira S. (2022). Radiation chemical yields of 7-hydroxy-coumarin-3-carboxylic acid for proton- and carbon-ion beams at ultra-high dose rates: Potential roles in FLASH effects. Radiat. Res..

[70] Iwata H., Ogino H., Hashimoto S., Yamada M., Shibata H., Yasui K., Toshito T., Omachi C., Tatekawa K., Manabe Y. (2016). Spot scanning and passive scattering proton therapy: Relative biological effectiveness and oxygen enhancement ratio in cultured cells. Int. J. Radiat. Oncol. Biol. Phys..

[71] Sørensen B.S., Sitarz M.K., Ankjærgaard C., Johansen J., Andersen C.E., Kanouta E., Overgaard C., Grau C., Poulsen P. (2022). In vivo validation and tissue sparing factor for acute damage of pencil beam scanning proton FLASH. Radiother. Oncol..

